# Deficiency of STING Promotes Collagen-Specific Antibody Production and B Cell Survival in Collagen-Induced Arthritis

**DOI:** 10.3389/fimmu.2020.01101

**Published:** 2020-06-03

**Authors:** Mookmanee Tansakul, Arthid Thim-uam, Thammakorn Saethang, Jiradej Makjaroen, Benjawan Wongprom, Trairak Pisitkun, Prapaporn Pisitkun

**Affiliations:** ^1^Section for Translational Medicine, Faculty of Medicine Ramathibodi Hospital, Mahidol University, Bangkok, Thailand; ^2^Center of Excellence in Systems Biology, Faculty of Medicine, Chulalongkorn University, Bangkok, Thailand; ^3^Division of Allergy, Immunology, and Rheumatology, Department of Medicine, Faculty of Medicine Ramathibodi Hospital, Mahidol University, Bangkok, Thailand

**Keywords:** STING, collagen-induced arthritis, autoantibody, B cell receptor, interferon

## Abstract

The levels of interferon-alpha are high in the serum and synovial fluid of rheumatoid arthritis (RA) patients. Activation of the stimulator of type I interferon genes (STING) mediates the productions of type I interferon and promotes chronic inflammation. STING plays a significant role in autoimmune lupus mice. However, the function of STING in collagen-induced arthritis (CIA) model has never been described. This study aimed to test the function of STING in CIA. The Sting-deficient mice developed arthritis comparable to WT mice. The levels of anti-collagen antibody from Sting-deficient mice were significantly higher than the WT mice. The B cells derived from Sting-deficient mice showed better survival than WT mice in response to the B cell receptor (BCR) stimulation. Activation of STING also induced B cell death, especially in activated B cells. This study demonstrated that the inhibition of STING promotes anti-collagen antibodies and B cell survival, which suggested that STING acts as a negative regulator of B cell function in the CIA model.

## Introduction

RA is a chronic systemic autoimmune disease that directly causes damage to the joints and cartilages and also induces inflammation throughout the body, such as the lung, eye, and cardiovascular system (1). The etiology of RA is unclear, and the response to the treatment is varied, which depends on numerous factors. The production of autoantibody against cartilage and joint could promote bone erosion and synovial hyperplasia (2, 3). Although several biologic drugs are available, not all patients can achieve remission or low disease activity (4). The successful treatment will prevent disability and improve the quality of life in RA patients.

The pathogenesis of rheumatoid arthritis (RA) is complicated and involves both innate and adaptive immunity (5). The synovium of RA contains two special cell types (fibroblast-like synoviocytes (FLS) and macrophage-like synoviocytes), which are part of innate immunity. These synoviocytes generate inflammatory mediators, including TNF-α, IL-1, IL-6, IL-17, IFN-γ, and chemokines that lead to synovial inflammation, bone erosion, and cartilage damage (6–8). The IL-17 signaling mediates the autoantibody production in the collagen-induced arthritis (CIA) model (9). However, the treatment response with an anti-IL17 monoclonal antibody in RA patients shows a high degree of heterogeneity (10). The inhibitors of the Janus kinase (JAK) pathway are approved for RA patients (11). These data suggest that the targeted multiple cytokines through the JAK pathway are useful for RA treatment.

Disturbance of type I IFNs (IFNs-I) signaling and production drive autoimmune development (12). The presymptomatic RA patients display an increase of IFNs-I before the onset of symptoms (13). The RA patients also show the elevation of IFN- α in the synovial fluid and high expression of IFNs-I regulated gene in peripheral blood mononuclear cells (PBMC) (14). However, the role of IFNs-I in arthritis and bone homeostasis has suggested the accelerating effect of arthritis and bone damage. The interferon-alpha receptor knockout mice develop arthritis severity higher than wild-type mice in the model of antigen-induced arthritis (15). IFNs-I also affects the bone homeostasis by inhibiting osteoclastogenesis via receptor activator of nuclear factor-kappa B (RANK) pathway, and reduction of c-FOS expression (16–18). Therefore, the goal of RA treatment with antagonizing the IFNs-I pathway has to be optimized between efficacy and potentially adverse effect.

STING is a cytosolic DNA sensor that initiates the production of IFNs-I. STING functions have been reported as both pro-inflammatory signaling and negative regulator against inflammation (19–21). The mutation in exon 5 of the *STING* gene results in gain function, leading to initiate inflammation, and cause the Sting associated vasculopathy with onset in infancy (SAVI) (22). Loss of STING function rescues *DNaseII*-deficient mice from lethality and polyarthritis (23). However, *Sting*-deficient lupus mice (MRL/Lpr mice) show higher and earlier mortality than *Sting*-sufficient MRL/Lpr mice. The *Sting*-sufficient MRL/Lpr mice showed an increase of lymphoid hypertrophy with inflammatory cell infiltration, autoantibodies, and cytokine production (24).

The objective of this study was to identify the role of STING in the pathogenesis of rheumatoid arthritis using collagen-induced arthritis (CIA) model as a representative model of the human RA.

## Materials and Methods

### Animals

The *Sting*^*gt*/*gt*^ mice were provided from Professor Paludan (Aarhus University, Denmark), while wild-type mice were purchased from the National Laboratory Animal Center, Nakornpathom, Thailand. The *Sting*^*gt*/*gt*^ is also known as the golden ticket (Tmem173gt) mice. *Sting*^*gt*/*gt*^ was created via chemically inducing mutagen with N-ethyl-N-nitrosourea (ENU) in the C57BL/6 background. The *Sting*^*gt*/*gt*^ mice carry a single nucleotide variant (T596A) of Sting, which led to undetectable STING protein by western blot (25). Mice were bred and housed in the facility at Faculty of Medicine, Chulalongkorn University, and all experiments were performed with the approval of the Animal Experimentation Ethics Committee of Chulalongkorn University Medical School with all relevant institutional guidelines.

### Collagen-Induced Arthritis (CIA) Model

The model was performed as previously described (26). CIA was induced in the mice at the age between 10 and 14 weeks. The immunization grade chick CII (2 mg/ml; #20012; Chondrex, Redmond, WA) was mixed gently with an equal volume of a 4 mg/ml Freund's complete adjuvant (CFA) (# 7001; Chondrex, Redmond, WA). Mice were anesthetized by isoflurane, and then intradermal injection was performed at multiple sites on the base of a tail (<50 μl/site). Experimental mice received 150 μl of CFA + CII emulsion, and control mice received only CFA or PBS. Three weeks after the first injection, mice received a booster injection of CFA + CII emulsion, CFA alone, or PBS. The mice were monitored and graded for arthritis severity (0 = normal; 1 = slight swelling and/or erythema; 2 = pronounced swelling; 3 = ankyloses) while performing the blind technique every other day.

### Detection of Anti-collagen Type 2 Specific Antibody

Chick collagen type 2 ELISA grade (#2011; Chondrex Redmond, WA) was coated on the plate overnight at 4°C. The plates were washed with 0.05% Tween-20 in PBS and then blocked with 2% BSA, 0.1% Tween-20 in PBS for 1 h at room temperature (RT). Then the plates were washed, added with the diluted serum, and incubated at 37°C for 1 h. Next, the plates were washed and added with antibodies to IgG (#115-035-146; Jackson ImmunoResearch Laboratories, PA, USA), IgG_2b_, IgG_2c_, or IgM conjugated with HRP (#1091-05, #1078-05 and #1021-05, respectively; SouthernBiotech, Birmingham, AL) were added and incubated for 1 h at 37°C. The plates were washed, added with OPD peroxidase substrate (Sigma-Aldrich, Darmstadt, Germany), and stopped reaction with 2N H_2_SO_4_. The absorbance was measured at 492 nm using the Varioskan Flash Microplate Reader (Thermo Fisher Scientific, MA USA).

The standard plate was coated with Goat Anti-Mouse IgM, IgG, IgG_2b_, or IgG_2c_ (#115-005-075, #115-005-146, #115-005-207 and #115-005-208, respectively; Jackson ImmunoResearch Laboratories, PA USA) and then serial dilutions of IgG (#015-000-003; Jackson ImmunoResearch Laboratories, PA, USA), IgG_2b_, IgG_2c_, or IgM (#0104-01, #0122-01 and #0101-01, respectively; Southern Biotech, Birmingham, AL) were added to obtain a standard linear curve. The intra-assay CV of ELISA testing for each of immunoglobulin isotypes is ranging from 3.74 to 6.97%.

### Histopathology

Mice were sacrificed 3 weeks after booster injection. Paws were fixed with 10% formalin, then decalcified with 10% ethylenediaminetetracetic acid (EDTA). The tissues were embedded in paraffin, sectioned, and stained with Haematoxylin and Eosin (H&E). The paw sections were viewed and captured under the Nikon Eclipse Ti-U microscope. Histology scores were graded and characterized by cell infiltration, synovial hyperplasia, and bone erosion (27). The qualitative scoring was evaluated with a blind technique.

### Flow Cytometry Analysis

Then the cell suspension was washed and stained for 15 min with antibodies as follows: B220 (RA3-6B2), CD3e (145-2C11), CD4 (GK1.5), CD45RB (C363-16A), CD11c (N418), CD11b (M1/70), CD95 (15A7), CD80 (16-10A1), and viability dye eFluor780 (Biolegend, San Diego, CA, USA). The stained cells were washed with 0.5% BSA in PBS and fixed with 1% paraformaldehyde in PBS. Sera from mice were collected at 3 weeks following the first immunization. Serum cytokines were measured by the LEGENDplex™ mouse inflammation panel kit (#740446; Biolegend, San Diego, CA, USA). Flow cytometry was performed using BD™ LSR-II (BD Biosciences, USA) and analyzed by FlowJo software (version 10, USA) and LEGENDplex™ Data Analysis Software.

### Single-Cell Isolation and B Cell Purification

Spleens and inguinal lymph nodes were collected. Every single cell was isolated by pressing spleens or lymph nodes through a cell strainer in cold 0.5% BSA in PBS solution. Red blood cells were lysed by adding cold ACK lysis buffer for 5 min. The cell suspension was washed and resuspended in 0.5% BSA in PBS. B cells were isolated from splenocytes by negative with anti-CD43 (Ly-48), which follows the protocol from the manufacturer (Miltenyi, Bergisch Gladbach, Germany).

### Microarray Analysis

RNA was isolated with Trizol reagent (Invitrogen, CA, USA) and followed by the RNeasy mini kit from Qiagen, MD, USA (catalog no. 74104). The RNA was labeled and hybridized using the Agilent One-Color Microarray-Based Gene Expression Analysis protocol (Agilent Technology, V 6.5, 2010). The results of the microarray were extracted with Agilent Feature Extraction software v11.0 (Agilent Technologies, Palo Alto, USA). We classified the gene function by using the online resource Database for Annotation, Visualization, and Integrated Discovery (DAVID, v6.8, https://david.ncifcrf.gov/), Interferome (http://www.interferome.org) and Reactome (https://reactome.org/) Microarray data was shown in the link below https://www.ncbi.nlm.nih.gov/geo/query/acc.cgi?acc=GSE142624.

### Proteomic Analysis

Dimethyl labeling and LC-MS/MS analysis were performed as described in Makjaroen et al. (28). In brief, B cells were isolated from Stinggt/gt and WT mice after injection with CFA and CII for 3 weeks. Isolated B cells were lysed with 5 %sodium deoxycholate (SDC) Thermo Fisher Scientific, MA USA) and trypsinized. Peptide 300 μg of WT and Stinggt/gt mice were labeled with light reagents) formaldehyde and cyanoborohydride) and medium reagents) formaldehyde-d2 and cyanoborohydride, respectively. The labeled peptides were pooled and fractionated into eight fractions before analysis with MS. The protein must be identified at least three samples from each group to qualify for further bioinformatics analysis. Peak intensity was calculated in log2 ratios L/M) and compared against a value of 0) no change, log2. Proteome Discoverer™ ver. 2.1 and MAXQUANT™ ver. 1.4.1.0 software was employed to quantify the relative MS signal intensities of dimethyl labeled-peptides. We classified the protein function by using the online resource Database for Annotation, Visualization, and Integrated Discovery DAVID, v6.8, https://david.ncifcrf.gov/ and Reactome https://reactome.org/. (The mass spectrometry proteomics data, including annotated spectra for all modified peptides and proteins identified based on a single peptide, have been deposited to the ProteomeXchange Consortium via the Proteomics IDEntifications PRIDE) partner repository with the data set identifier PXD018652.

### *In vitro* Stimulation Assay

In brief, B cells (1 × 10^5^ cells) were incubated with 10 μg/ml of full-length IgG or F(ab')_2_ fragment (#115-005-075 and #115-006-02; Jackson ImmunoResearch Laboratories, PA, USA) in 10%FCS/RPMI with the supplement of 1X MEM non-essential amino acid, 1 mM Na pyruvate, 2 mM L-glutamine, 10 mM Herpes, 100 u/ml of Pen/Strep, and 0.05 mM 2-mercaptoethanol for 48 h at 37C and 5% CO_2_. The proliferation of B cells was examined by the MTS assay followed by the manufacturer protocol (#G3580, Promega Corporation, Madison, USA).

The viability of B cells was examined after the stimulation with DMXAA (5,6-Dimethylxanthenone-4-acetic acid or STING ligand). In brief, B cells (1 × 10^6^ cell/ml) were cultured in 6-well-plates and then stimulated with 10 μg/ml DMXAA (Invivogen, San Diego, USA) and DMSO (control group) for 6 and 24 h at 37°C and 5% CO_2_. Then B cells were stained with antibody and Fixable Viability Dye eFluor® 780.

### CFSE Labeling

CFSE (Carboxyfluorescein succinimidyl ester) labeled B cell analysis was described using the protocol below. The 1 × 10^6^ B cells in pre-warmed PBS were incubated with 0.5 uM CFSE (Biolegend, San Diego, CA, USA) in the CO_2_ incubator at 37°C for 10 min. The labeling activity was quenched by adding 10 volumes of culture medium. Then, labeled cells were centrifuged at 4°C with a speed of 1,500 rpm for 5 min. After centrifugation, the supernatant was discarded, and cells were re-suspended in the culture medium.

### Statistical Analysis

The sample size of each experiment was calculated by the G-Power software version 3.1.9.2. The statistical test in this study was performed with Excel 2016, GraphPad Prism version 5 (for window), and also Instant Clue software. The data was evaluated normality by the Shapiro-Wilk test. Independent *t*-tests were used in case of the normal distribution; otherwise, the Mann–Whitney *U*-test was applied. The significant statistical change was considered when *P* < 0.05. The arthritis scores were analyzed with multiple comparisons using Friedman's Two-way Anova for every time points of all groups, and the results of the comparisons were statistically significant (*P* < 0.001). We then performed *post-hoc* analysis by the Mann-Whitney correction of all pairwise. We performed the multiple testing correction using Bonferroni correction of the *P*-values.

For the proteomic analysis, protein must be identified at least three from each mice group (If the protein did not pass this criterion, the protein must be excluded from downstream analysis). A hierarchical cluster was used to analysis of heatmap (Euclidean metric and complete linkage). The heatmap and volcano plot was operated by Instant Clue software.

## Results

### Sting Was Not Necessary for Arthritis Development in the CIA Model

**Figure 1:**
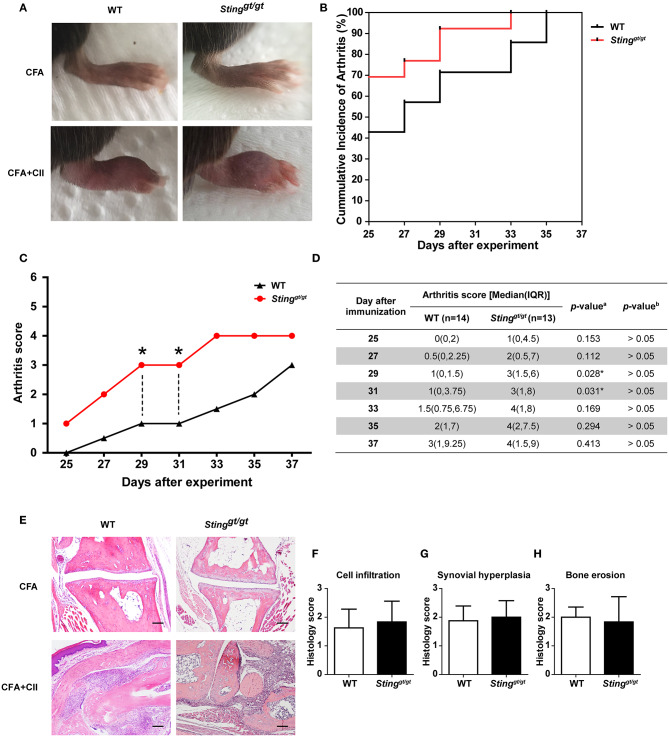
STING was not necessary for arthritis development in the CIA model. The mice were injected with CFA or CFA plus chicken collagen type 2 on days 0 and 21. The clinical sign of arthritis and pathology were observed. **(A)** The representative of hind paws from WT and *Sting*^*gt*/*gt*^ mice, and **(B)** the cumulative incidence of arthritis from WT and *Sting*^*gt*/*gt*^ mice are shown. **(C,D)** Arthritis scores were blindly graded every other day. Data are shown as the median on **(C)** line chart and **(D)** interquartile range (*p < 0.05; a = Mann-Whitney *U*-test; b = Bonferroni correction; *N* = 13–14 mice/group). **(E)** Histopathology of hind paws was stained with H&E; Scale bar = 100 um. Histology scores were graded with blind technique on three parameters, **(F)** cell infiltration, **(G)** synovial hyperplasia, and **(H)** bone erosion. Data are shown as mean ± SEM; *N* = 3 mice/group.

Both WT and *Sting*^*gt*/*gt*^ mice developed clinical arthritis within Day 42 (6th week) after the initial immunization (Figure 1A). The accumulative incidence of arthritis was comparable between WT and *Sting*^*gt*/*gt*^ mice (Figure 1B). We observed that the *Sting*^*gt*/*gt*^ mice showed a significantly higher value in arthritis score than WT mice on Day 29 and 31 (Figures 1C,D). However, the multiple comparisons by Bonferroni correction did not demonstrate the difference in arthritis score (adjusted *P* > 0.05; Figure 1D). The histopathology showed the similarity in inflammatory cell infiltration, synovium hyperplasia, and bone erosion between WT and *Sting*^*gt*/*gt*^ mice (Figures 1E–H). These data suggested that STING was not required for arthritis development in the CIA model.

### Immunophenotypes in Response to CIA

**Figure 2:**
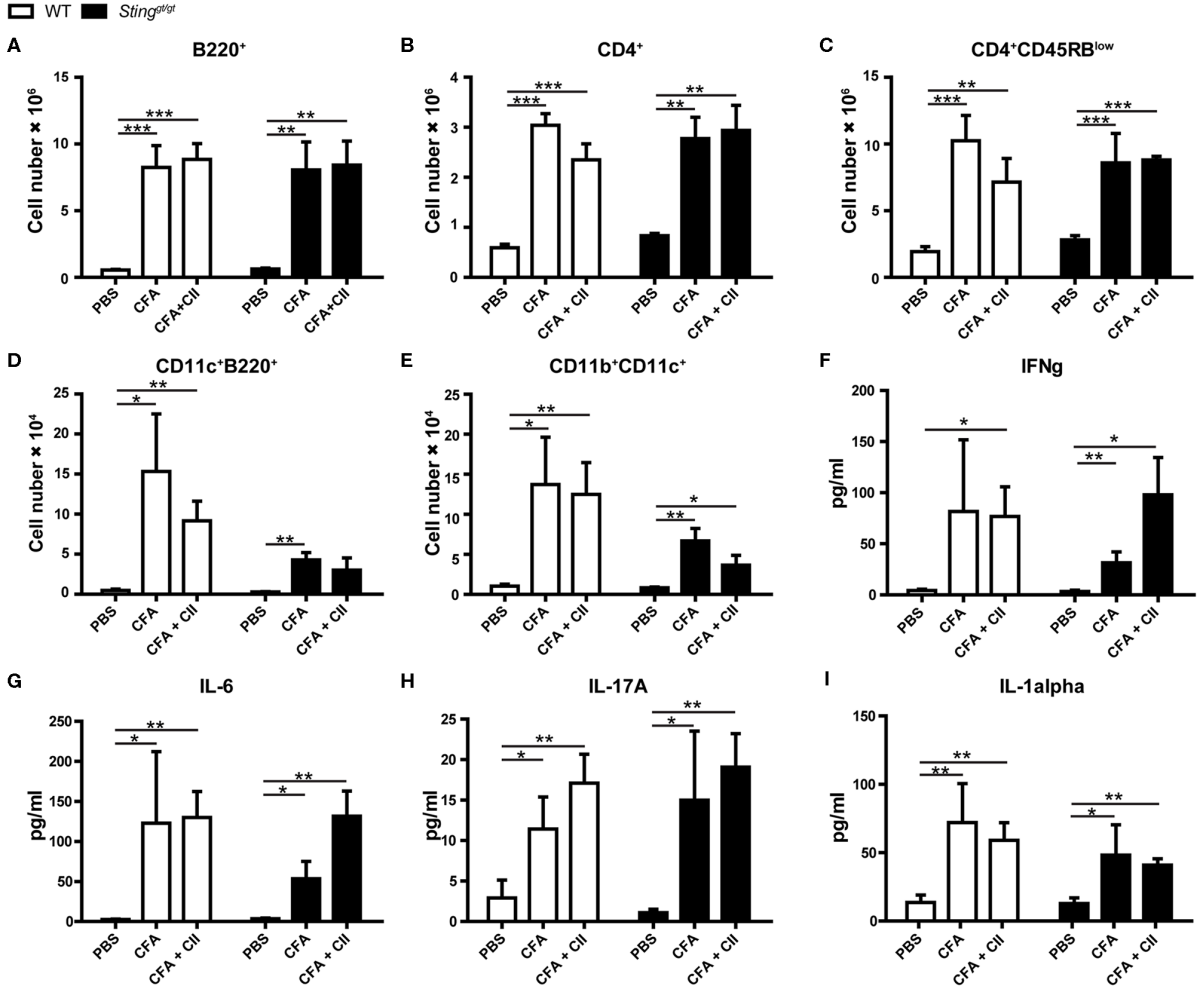
Immunophenotypes in response to CIA. The samples were collected from WT and *Sting*^*gt*/*gt*^ mice after the 1st immunization with CFA, CFA + CII, or PBS control for 3 weeks. **(A–E)** Flow cytometry analysis of inguinal lymph nodes (*N* = 4–7 per group) are shown in the number of **(A)** B220^+^ cells, **(B)** CD4^+^ cells, **(C)** CD4^+^CD45RB^low^ cells, **(D)** CD11c^+^B220^+^ cells (pDC), **(E)** CD11b^+^CD11c^+^ (mDC). **(F–I)** The serum cytokines of **(F)** IFN-γ, **(G)** IL-6, **(H)** IL-17A, and **(I)** IL-1α were measured. Data are shown as mean ± SEM; *N* = 3–8 mice for PBS control or CFA; *N* = 10–12 mice for CFA+CII (*p < 0.05, **p < 0.01, and ***p < 0.001).

To identify the immunophenotypes that mediated through the STING signaling pathway, we analyzed the flow cytometry of the isolated cells from draining lymph nodes (LN) of the mice after the first immunization for 3 weeks. We found that the number of B220^+^ cells (Figure 2A), CD4^+^ cells (Figure 2B), CD4^+^CD45RB^low^ (Figure 2C), CD11c^+^B220^+^ (Figure 2D), and CD11b^+^CD11c^+^ (Figure 2E) were increased in the CFA controls and CFA+CII treatment compared to PBS injection. These changes in the LN similarly occurred in both WT and *Sting*^*gt*/*gt*^ mice. Also, we detected the inflammatory cytokines in the serum of both WT and *Sting*^*gt*/*gt*^ mice and did not see the difference in response to the first immunization. The levels of IFN-γ (Figure 2F), IL-6 (Figure 2G), IL-17A (Figure 2H), and IL-1α (Figure 2I) increased in both CFA and CFA + CII. These data suggested the STING did not influence of the immunophenotypes that responded to the CFA or CFA + CII in the draining LN and inflammatory cytokines that we tested in the serum.

### The Sting-Deficient Mice Developed Higher Levels of Anti-collagen Type 2 Antibodies

The arthritis score did not show the statistically significant between WT and STING-deficient mice when using multiple corrections (Figure 1D), which could result from the number of mice in the experiments. However, we observed some of the STING-deficient mice developed arthritis earlier than WT mice. Thus, we questioned whether STING might act as a negative regulator to control the initial process of autoimmune arthritis in the CIA model.

**Figure 3:**
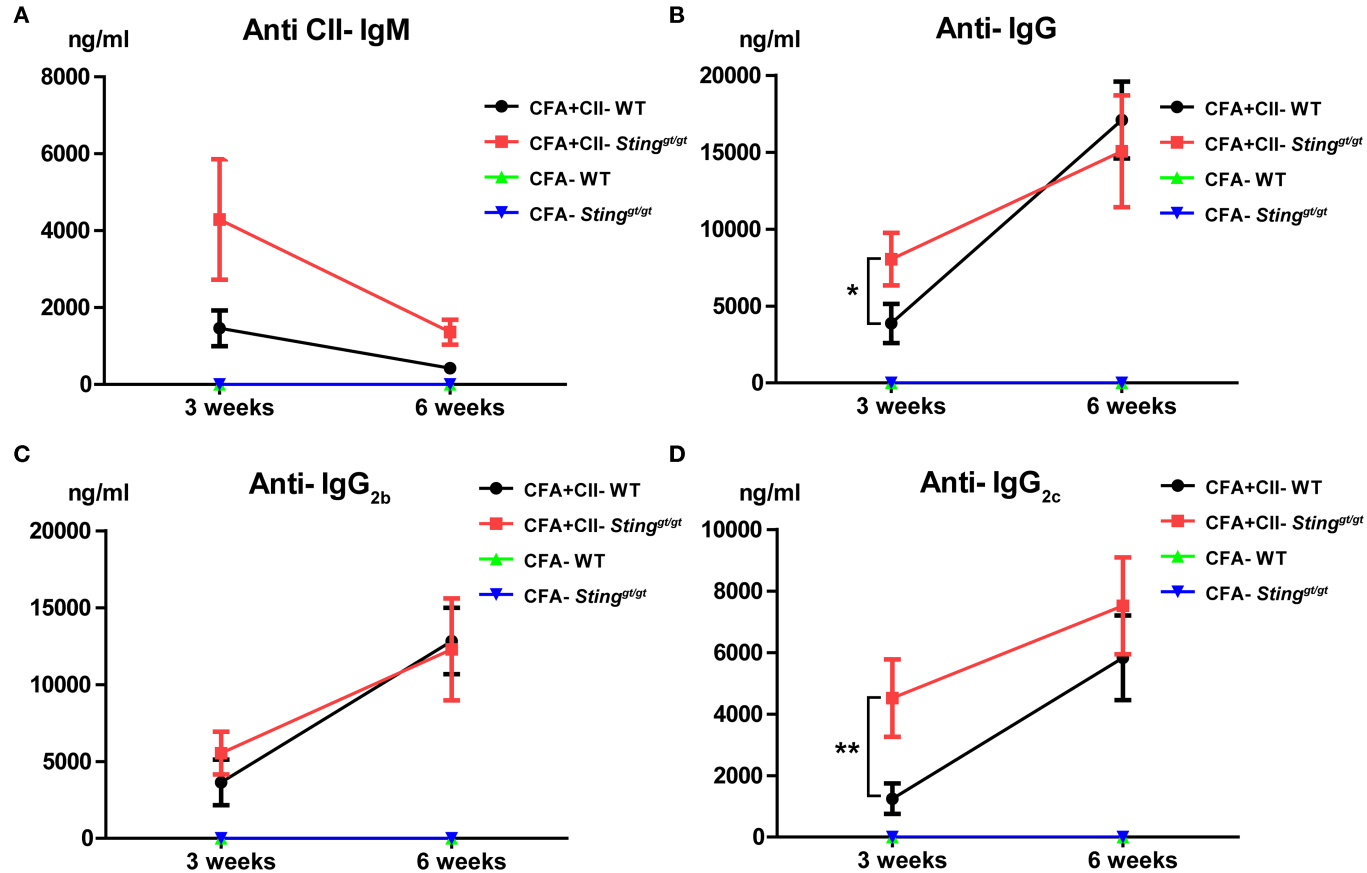
The STING-deficient mice developed a higher production of anti-collagen type 2 antibody. The level of anti-collagen type 2 antibodies detecting by the ELISA technique after the 1st immunization for 3 weeks, and the 2nd immunization for 3 weeks are shown on **(A–D)**. The isotypes of anti-collagen type 2 antibodies in **(A)** IgM, **(B)** IgG, **(C)** IgG_2b_, and **(D)** IgG_2c_ are shown in concentration as mean ± SEM; *N* = 5 mice/group in CFA control and *N* = −11 mice/group in CFA+CII treatment (*p < 0.05 and **p < 0.01).

Anti-collagen type 2 antibodies are pathogenic and mediate arthritis in the collagen antibody-induced arthritis (CAIA) (29). We hypothesized that the STING-deficient mice produced the anti-collagen type 2 (CII) antibodies higher than WT mice, which led to the early development of arthritis. The level of anti-CII antibodies detected by ELISA showed no statistically significant difference of anti-CII IgM after 3 and 6 weeks of the first and second injection of CFA and CII (Figure 3A). However, we detected the significant difference of anti-CII IgG and IgG_2c_ after 3 weeks of CFA and CII injection (Figures 3B,D) while anti-CII IgG_2b_ slightly showed a trend higher in the STING-deficient mice (Figure 3C). Interestingly, the anti-CII IgG and isotypes showed a comparable amount after the second boost with CFA and CII (Figures 3B,D). These data suggested that STING may be involved in the initiation of autoantibody production in the CIA model.

### Gene Expression Profiles of Splenocytes in Response to CIA

**Figure 4:**
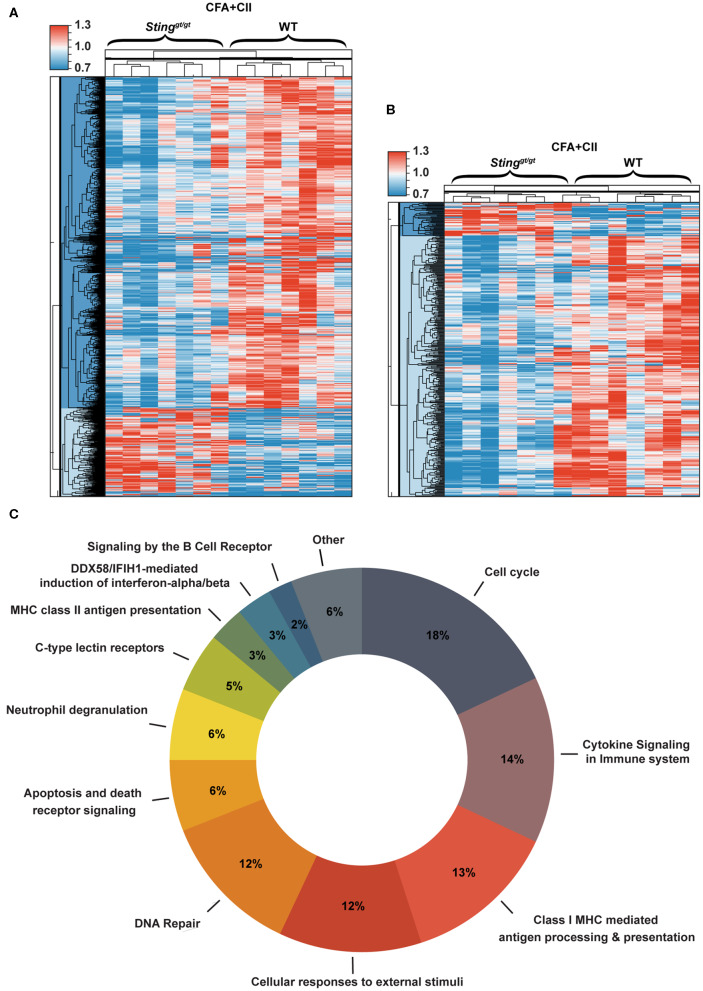
Gene expression profiles of splenocytes in response to CIA. Gene expression profiles that significantly changed in the isolated splenocytes from WT and *Sting*^*gt*/*gt*^ mice after the 1st immunization with CFA + CII for 2 weeks were analyzed. **(A,B)** The heat map shows the genes that significantly changed comparing between WT and *Sting*^*gt*/*gt*^ mice (*p* < 0.05). **(A)** Data of all of the genes and **(B)** the interferon signature genes are shown in the sample signal/average signal (*N* = 7 mice per group). **(C)** The bioinformatics analysis of microarray data shows the molecular function of genes that significantly different between WT and *Sting*^*gt*/*gt*^ mice after the immunization with CFA + CII for 2 weeks.

To globally identify the biological process that changed in the immune system after the CIA, we isolated splenocytes from WT and *Sting*^*gt*/*gt*^ mice after the 1st immunization with CFA + CII for 2 weeks and analyzed gene expression profiles. The gene expression profiles that were significantly different between WT and *Sting*^*gt*/*gt*^ mice are shown in the heat map (Figures 4A,B). The changes in gene expression profiles of splenocytes included both interferon-inducible genes and non-related interferons (Figure 4A). Most of the interferon-related genes were down-regulated in the *Sting*^*gt*/*gt*^ mice (Figure 4B). These data suggested that the severity of CIA in *Sting*^*gt*/*gt*^ mice may not directly be related to the interferon signaling pathway. Next, we analyzed the molecular function of these gene lists. Interestingly, the biological process related to the signaling by the B cell receptor (BCR) showed up in the data of isolated splenocytes (Figure 4C). The lists of biological processes were shown (Supplementary Table 1). These data pointed out the potential function of STING signaling in B cell biology.

### Sting Negatively Regulates BCR Induced Cell Proliferation

**Figure 5:**
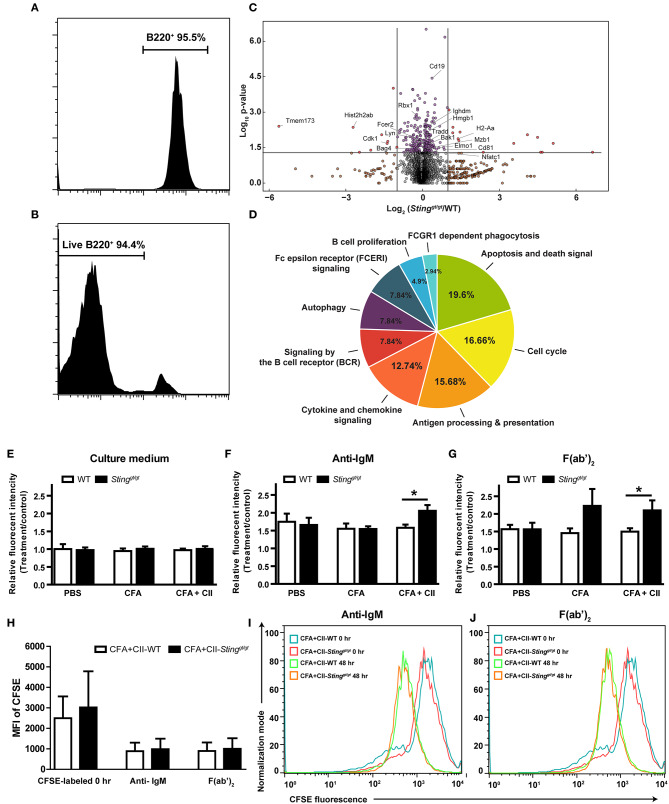
STING negatively regulates B cell survival. Isolated B cells from the spleens of immunized mice (WT and *Sting*^*gt*/*gt*^) with CFA+CII were analyzed by LC-MS/MS **(A–D)**. **(A)** The isolation of B cells shown up to 95% of purity, and **(B)** the B cells had 94% viability that was subjected to B cell functional study. **(C)** The data from LC-MS/MS experiment shown in the volcano plot (*N* = 6 samples per group, and each sample consist of 2 mice; *p* < 0.05). **(D)** The pie chart shows the bioinformatics analysis of the proteins that characterized the molecular functional groups. **(E–J)** Isolated B cells from the spleens of WT and *Sting*^*gt*/*gt*^ mice after the injection with PBS, CFA, or CFA + CII were incubated with **(E)** culture medium, **(F)** anti-IgM, and **(G)** F(ab')_2_ for 48 h then measured the cell viability by the MTS assay. **(H–J)** CFSE dilution assay of B cells after stimulation with anti-IgM, and F(ab')_2_ to determine the proliferation. **(H)** The mean fluorescence intensity (MFI) of CFSE labeling B cells are shown as mean ± SEM; *N* = 3–4 mice/group (*p < 0.05). The representative histograms of CFSE labeling B cells isolated from WT and *Sting*^*gt*/*gt*^ mice after stimulation with **(I)** anti-IgM and **(J)** F(ab')_2_ are shown.

To identify the function of STING in B cells, we isolated the B cells from the spleen of immunized mice with CFA + CII and achieved up to 95% of the purity (Figure 5A). The viability of the isolated B cells was more than 90% (Figure 5B). These B cells were subjected to analysis by LC-MS/MS. The volcano plot shows the proteins that were changed after CFA + CII comparing between WT and *Sting*^*gt*/*gt*^ mice (Figure 5C). The proteins that showed the difference in expression were analyzed and categorized. The pie chart illustrates the biological processes that occurred in B cells (Figure 5D). The molecular functions that changed were B cell proliferation, B cell receptor signaling, antigen presentation, and death signal (Figure 5D). The bioinformatics analysis of protein expression in B cells suggested that STING may be involved in B cell signaling and B cell proliferation. The lists of biological processes were shown (Supplementary Table 2).

To test if the B cell signaling mediated through STING, we stimulated the isolated B cells from the WT and *Sting*^*gt*/*gt*^ mice that were injected with PBS control, CFA, or CFA + CII (Figures 5D–G). The B cells from all of the treatments that were cultured in the regular medium showed no proliferation (Figure 5E). The anti-IgM stimulated B cells to proliferate in PBS control and CFA injected groups from both WT and *Sting*^*gt*/*gt*^ mice measured with MTS assay (Figure 5F). However, the B cells from the CFA + CII treatment group showed the difference in MTS fluorescence intensity between WT and *Sting*^*gt*/*gt*^ mice (Figure 5F). The B cells from *Sting*^*gt*/*gt*^ mice significantly showed the MTS fluorescence intensity higher than WT mice (Figure 5F). A similar pattern of B cell response was detected when stimulated with F(ab')_2_ (Figure 5G). The difference of fluorescence intensity from MTS assay could derive from the change of proliferation or the viability of B cells. Thus, we performed the CFSE dilution assay to determine whether the proliferation or the viability that was mediated by STING activation in B cells. The stimulation with anti-IgM and F(ab')_2_ induced the CFSE dilution assay of B cells isolated from WT and *Sting*^*gt*/*gt*^ mice in a similar manner (Figures 5H–J). These data suggested that STING played a role as a negative regulator of B cell survival in the stimulated B cells.

### Activation of Sting Mediated B Cell Death

**Figure 6:**
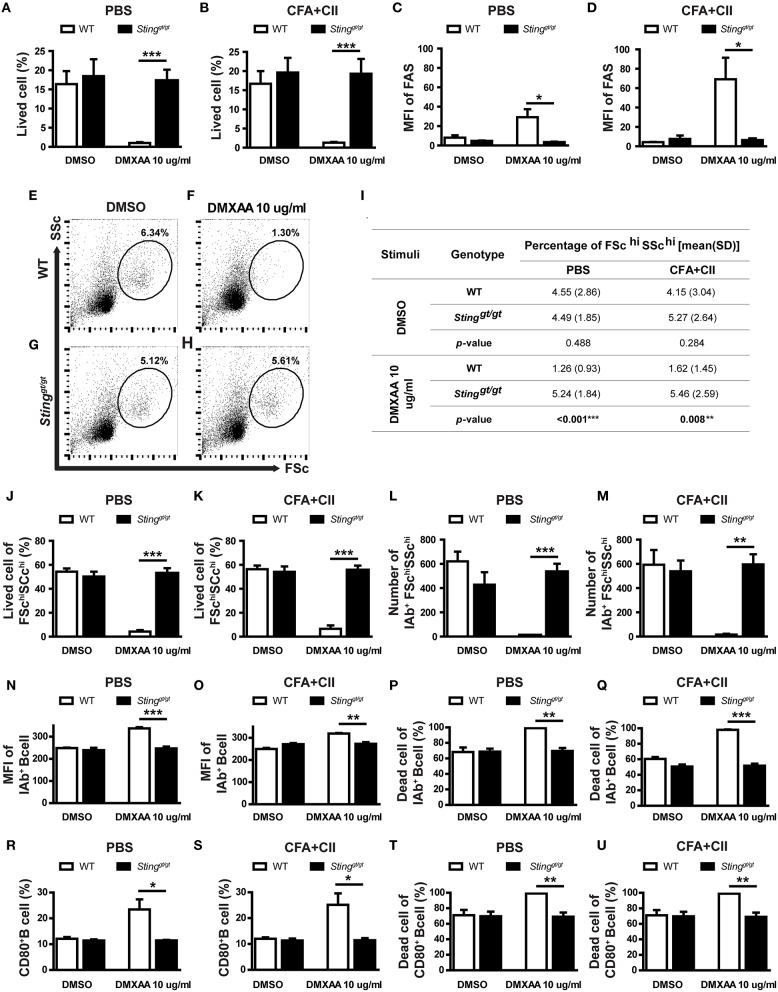
Activation of STING mediated B cell death. The isolated B cells from the spleens of immunized mice (WT and *Sting*^*gt*/*gt*^) were cultured with DMSO control or DMXAA for 24 h and analyzed by flow cytometry. **(A,B)** Percentage of B cell viability of **(A)** PBS and **(B)** CFA+CII injection group. **(C,D)** Mean fluorescence intensity (MFI) of FAS on B cells from **(A)** PBS and **(B)** CFA+CII injection group. **(E–H)** Representative plots from the PBS injection group show FSC and SSc. The gated areas show the FSc^hi^SSc^hi^ B cells population. The isolated B cells from WT and *Sting*^*gt*/*gt*^ mice were cultured with **(E,G)** DMSO and **(F,H)** DMXAA. **(I)** Data showed the percentage of FSc^hi^SSc^hi^ B cells isolated from the immunized WT and *Sting*^*gt*/*gt*^ mice after incubation with DMSO and DMXAA. **(J–U)** Flow cytometry analysis showed **(J,K)** the percentage of viable cells in FSc^hi^SSc^hi^ B cells and **(L,M)** the number of IAb^+^FSc^hi^SSc^hi^ B cells from the mice injected with PBS and CFA+CII. **(N,O)** Mean fluorescence intensity (MFI) of IAb^+^ on B cells from **(N)** PBS and **(O)** CFA + CII injection group. **(P,Q)** Percentage of dead IAb^+^ B cells of the mice injected with **(P)** PBS and **(Q)** CFA + CII. **(R,S)** Percentage of CD80^+^ B cell from **(R)** PBS and **(S)** CFA+CII injection group. **(T,U)** Percentage of dead CD80^+^ B cells of the mice injected with **(T)** PBS and **(U)** CFA+CII. Data are shown as mean ± SEM; *N* = 3-6 mice/group (*p < 0.05, **p < 0.01, and ***p < 0.001).

Our bioinformatics analysis showed the alteration of the apoptosis and death pathway that may be related to STING signaling (Figures 4C, 5D). Also, our data suggested that STING may affect the survival of B cells (Figures 5F–H). To confirm that STING can mediate cell death in B cells, the isolated B cells from the spleen of WT and *Sting*^*gt*/*gt*^ mice were incubated with DMXAA (STING ligand) and subsequently analyzed by flow cytometry. The B cells from the mice that were treated with PBS or CFA + CII did respond to DMXAA in a similar pattern (Figures 6A,B). The STING activation mediated B cell death in WT mice but not in *Sting*^*gt*/*gt*^ mice (Figures 6A,B). We also looked at the mean fluorescence intensity (MFI) of FAS on B cells and found an increase in FAS expression upon DMXAA stimulation (Figures 6C,D). These data suggested that STING mediated B cell death and increased FAS expression in both naïve and antigenic primed B cells.

The analysis by flow cytometry showed the absence of FSc^hi^SSc^hi^ B cells in WT mice after STING activation, while *Sting*^*gt*/*gt*^ mice showed the presence of these B cell subsets (Figure 6E). The quantification of these B cell subsets was shown (Figure 6I). The activation with DMXAA significantly reduced the percentage of these FSc^hi^SSc^hi^ B cell subsets in WT mice (Figure 6I). These data suggested that STING mainly targeted these FSc^hi^SSc^hi^ B cell subsets. Next, we determined whether the absence of these B cell subsets after STING activation in WT mice resulted from cell death. We detected the percentage of viable cells in the FSc^hi^SSc^hi^ B cells was significantly decreased in WT mice after STING activation but was preserved in *Sting*^*gt*/*gt*^ mice (Figures 6J,K). The FSc^hi^SSc^hi^ B cells may represent the activated B cells, which were the preferred target of STING mediated cell death. Further analysis showed that the number of IAb^+^ cells in the FSc^hi^SSc^hi^ B subset significantly diminished in WT mice upon STING activation (Figures 6L,M). The activation of STING mediated B cell survival was detected in the first 6 h as well (Supplementary Figure 1).

Next, we confirmed that STING mediated B cell death was targeted at the activated B cell by staining CD80 (a costimulatory molecule) and IAb (MHC-II) as a marker of the activated B cells. The MFI of IAb and percentage of CD80^+^ B cells significantly increased in the DMXAA activated WT B cells compared to *Sting*^*gt*/*gt*^ B cells (Figures 6N,O,R,S). Also, the percentage of dead cells in IAb^+^ B cells and CD80^+^ B cells significantly increased in WT B cells but not in *Sting*^*gt*/*gt*^ B cells after DMXAA stimulation (Figures 6P,Q,T,U). These results suggested that STING activated B cell death.

## Discussion

Collagen-Induced arthritis (CIA) model has similar features with rheumatoid arthritis (30). The immunization with complete Freund's adjuvant (CFA) and collagen type 2 induces the production of anti-collagen type 2 antibody, rheumatoid factor (RF), and anti-cyclic citrullinated peptide antibodies (anti-CCP) (30). The scores of arthritis in the *Sting*^*gt*/*gt*^ mice that were comparable to WT mice in this study were unexpected. We detected the significant reduction of CD11c^+^B220^+^ and CD11b^+^CD11c^+^ cells in the *Sting*^*gt*/*gt*^ mice after the CIA, which may suggest that STING functioned in the promotion of dendritic cells. However, the differnce of antigen-presenting cells (APC) between WT and *Sting*^*gt*/*gt*^ mice did not explain the similarity in arthritis phenotypes of both WT and *Sting*^*gt*/*gt*^ mice in the CIA.

The adaptive immunity is crucial for CIA development. The *CD4*-deficient mice are less susceptible to the CIA than WT mice (31). Th17 cells were shown as one of the significant players of the CIA (32). The IFN-γ knockout mice increase the Th17 cells which accelerate the CIA (33). STING activation in T cells induces the production of IFN-I and IFN-γ (34). However, the *Sting*^*gt*/*gt*^ and WT mice showed a similar pattern of serum IFN-γ and IL-17 in response to the CIA. Also, our study did not detect the difference in T cell phenotypes between WT and *Sting*^*gt*/*gt*^ mice. The data suggested that the difference in arthritis between WT and *Sting*^*gt*/*gt*^ mice did not contribute by T cell alteration.

The B cells are required for CIA development (35). The rise of anti-collagen type 2 antibody in the early phase in the *Sting*^*gt*/*gt*^ mice, before the second immunization, suggested the increase of B cell activity in the *Sting*^*gt*/*gt*^ mice. Although the severity of arthritis was caught up with WT mice at week 6, this finding may be explained by the fact that the levels of anti-collagen type 2 antibody were boosted after the second immunization with CFA and CII. Interestingly, the analysis of gene expression profiles from splenocytes at the early phase after the first immunization suggested the increase in B cell signaling. Also, the expression of interferon-inducible genes decreased in the *Sting*^*gt*/*gt*^ mice compared with WT after the CIA. The administration of IFN-β into the CIA mice reduced inflammation and slow cartilage destruction (36). The combination of the increase of B cell activity and the decrease in IFN-I function may promote arthritis development in the *Sting*^*gt*/*gt*^ mice.

The function of STING in B cells showed different results. The expression of STING on B cell is required for antibody response to T cell-dependent antigens and cyclic-di-GMP (CDG) (37). The activation of STING with CDG acts synergistically with B cell receptors to increase the expression of co-stimulatory molecules (CD86) (37). The STING-deficient MRL/lpr mice developed severe autoimmunity with an increase in autoantibody production (24). Another study shows that STING negatively regulates dsDNA-activated JAK1-STAT1 via SHP-1/2 in B cells (38). SHP-1 is a protein-tyrosine phosphatase that dephosphorylates the proximal BCR signaling or mediates the function of certain inhibitory ITIM containing receptors (39). The deletion of SHP-1 in B cells leads to systemic autoimmunity (40). Here, we detected the increase of B cell survival in the *Sting*^*gt*/*gt*^ mice upon BCR engagement with anti-IgM and F(ab')_2_, especially in the isolated B cells from mice that were immunized with CFA and collagen type 2.

The CD43^−^ B cells are immature follicular B cells (41). In theory, these populations should not be different between immunized or non-immunized mice. Our data showed the difference in BCR response of CD43^−^ B cells from the immunized group (CFA+CII) of STING-deficient mice. However, the CD43 (or S7) negative cells upregulate the expression of CD43 after LPS stimulation (42). Due to the CD43 negative selection it is possible that antigen specific B cells in our *in vitro* experiments only comprised a fraction of all antigen activated B cells. These data suggested that STING worked as a negative regulator for BCR signaling in the stimulated B cells.

The proteomics data of B cells showed the increase of several proteins involved in apoptosis and death signals. Activation of STING induces apoptosis in malignant B cells (43). We found that STING ligand-induced B cell death, which broadly affected either naïve or stimulated B cells. The flow cytometry showed that the preferentially targeted B cells for STING induced cell death were the activated B cells (IAb^+^FSC^hi^SSc^hi^, or CD80^+^). Thus, the absence of STING in B cells maintained the activated B cell to survive and allowed the stimulated B cells to respond to the signaling from BCR, which subsequently augmented the antibody production and antigenic presentation.

In summary, DNA sensing through the STING pathway activates innate immunity and induces inflammation (19). Our study demonstrated that STING promoted the expression of IFN-inducible genes and dendritic cell expansion in the CIA. At the same time, STING functioned as a negative regulator in the B cells upon BCR engagement, a mechanism that controls the hyperactivation of B cells. STING-deficient mice do not spontaneously develop autoimmune diseases (44). These data suggested the STING worked differently in a particular cell type with a specific stimulation. The counter-regulatory function of STING in the adaptive immunity could keep the balance between inflammation and tolerance.

## Conclusion

Inhibition of STING in B cells promoted the activated B cell survival in the CIA model.

## Data Availability Statement

The datasets generated for this study can be found in the the link below, Microarray; https://www.ncbi.nlm.nih.gov/geo/query/acc.cgi?acc=GSE142624. The GEO accession number is GSE142624. The mass spectrometry data, including annotated spectra for all modified peptides and proteins identified based on a single peptide, have been deposited to the ProteomeXchange Consortium via the Proteomics IDEntifications (PRIDE) partner repository with the data set identifier PXD018652.

## Ethics Statement

The animal study was reviewed and approved by Animal Experimentation Ethics Committee of Chulalongkorn University Medical School.

## Author Contributions

MT performed experiments, interpreted data, conceptualization, and writing manuscript. AT, BW, and JM provided methodology and formal analysis. TS provided formal analysis and data curation. TP contributed funding acquisition, conceptualization, methodology, and editing manuscript. PP provided conceptualization data curation, formal analysis, funding acquisition, and writing manuscript.

## Conflict of Interest

The authors declare that the research was conducted in the absence of any commercial or financial relationships that could be construed as a potential conflict of interest.
