# Supplementary material for: Deficiency of STING Promotes Collagen-Specific Antibody Production and B Cell Survival in Collagen-Induced Arthritis
Source: Front Immunol. 2020 Jun 3;11:1101. doi: 10.3389/fimmu.2020.01101 (PMC7283782; doi:10.3389/fimmu.2020.01101)
Supplement: Supplementary file 1 [file Table_1.DOCX]

**Supplement Table 1: Biological process of RNA expression from Microarrays data**

| **Biological process** | **Up in *Sting^gt/gt^*** | **Down in *Sting^gt/gt^*** | **Number** |
| --- | --- | --- | --- |
| **Cell Cycle** | Nupl2, Top3a | Anapc1, Casc5, Ccnh, Cdc23, Cdkn1b, Cep192, Dido1, Dync1li2, Ep300, Esco1, Fbxw11, Haus6, Kif23, Lin9, Lpin2, Mcph1, Mpp2, Ncapd2, Nup133, Nup160, Orc5, Pds5a, Phf8, Pot1b, Psmf1, Tmpo, Top2a, Topbp1, Tubgcp3 | 31 |
| **Cytokine Signaling in Immune system** | Camk2a, Camk2b, Mid1 | Arih1, Atf2, Bcl2l1, Fbxw11, Gsta2, H2-M5, Hspa8, Ifnar1, Il1rl1, Irf9, Isg20, Nup133, Nup160, Sos1, Tab2, Trim25, Ube2l6, Zeb1 | 24 |
| **Class I MHC mediated antigen processing & presentation** | Fbxo10, Rnf217, Trim50 | Ccnf, Cdc23, Cul3, Fbxl20, Fbxl3, Fbxw11, Fbxw2, Gan, H2-M5, Hace1, Herc1, Herc6, Klhl25, Lrr1, Pja2, Psmf1, Trim69, Trip12, Ube2h, Ube2l6 | 23 |
| **Cellular responses to external stimuli** | Id1, Nupl2 | Anapc1, Arnt, Atg4a, Cat, Cdc23, Cdkn1b, Crebbp, Dnajb1, Dync1li2, Ep300, Hspa1b, Hspa8, Nup133, Nup160, Pot1b, Psmf1, Rb1cc1, Sirt1, Tnrc6b, Wipi1 | 22 |
| **DNA Repair** | Mid1, Rad52, Top3a | Cat, Ccnh, Ep300, Ercc6, Mbd4, Neil3, Pms2, Polh, Ppp4r2, Rad23a, Rif1, Tcea1, Tdp2, Topbp1, Trim25, Ube2l6, Usp45, Uvssa, Wdr48 | 22 |
| **Neutrophil degranulation** |  | Cat, Cdk13, Dnase1l1, Golga7, Grn, Hspa1b, Hspa8, Rap1a, Rock1, Tmc6 | 10 |
| **C-type lectin receptors (CLRs)** | Card9, Muc19 | Crebbp, Ep300, Fbxw11, Nfatc3, Psmf1, Tab2 | 8 |
| **Apoptosis** | Creb3l3 and Tiam2 | Akap13, Arhgef39, Atf4, Bcl2l1, Crebbp, Ect2, Itsn1, Nfatc3, Psmf1, Rock1, Sos1 | 6 |
| **MHC class II antigen presentation** |  | Dync1li2, H2-M5, Kif11, Kif15, Kif5b | 5 |
| **DDX58/IFIH1-mediated induction of interferon-α/β** |  | Crebbp, Ep300, Otud5, Trim25, Ube2l6 | 5 |
| **Death receptor signaling** | Creb3l3 and Tiam2 | Akap13, Arhgef39, Ect2, Itsn1, Sos1 | 5 |
| **Signaling by the B Cell Receptor (BCR)** |  | Fbxw11, Nfatc3, Psmf1, Sos1 | 4 |
| **TGF signalling** |  | Rap1a, Tab2, Wipf2 | 3 |
| **Nucleotide-binding domain, leucine rich repeat containing receptor (NLR) signaling pathways** | Card9 | Bcl2l1, Tab2 | 3 |
| **Immuno-regulatory interactions between a Lymphoid and a non-Lymphoid cell** | Trem2 | Colec12 | 2 |
| **Cytosolic sensors of pathogen-associated DNA** |  | Crebbp and Ep300 | 2 |
