# Supplementary material for: Deficiency of STING Promotes Collagen-Specific Antibody Production and B Cell Survival in Collagen-Induced Arthritis
Source: Front Immunol. 2020 Jun 3;11:1101. doi: 10.3389/fimmu.2020.01101 (PMC7283782; doi:10.3389/fimmu.2020.01101)
Supplement: Supplementary file 2 [file Table_2.DOCX]

**Supplement Table 2: Biological process of B cell protein from LC-MS/MS**

| **Biological process** | **Up in *Sting^gt/gt^*** | **Down in *Sting^gt/gt^*** | **Number** |
| --- | --- | --- | --- |
| **Apoptosis and death signal** | Aimp2, Bak1, Ctnnbl1, Dpf2, Elmo1, Fis1, Gpx1, Lamtor1, Ogt, Prkacb, Pygb, Rad21, Stk10 and Tradd | Bag4, Ccar2, Cdk1, Huwe1, Noc2l and Thoc1 | 20 |
| **Cell cycle** | Golga2, Hist2h2be, Numa1, Ppp1cb, Psma3, Rad21, Rad9a, Sec13 and Ube2v2 | Cdk1, Dync1li1, Psmd13, Psmd14 Rangap1, Rbbp4, Rbx1 and Rfc3 | 17 |
| **Class I MHC mediated antigen processing & presentation** | Lrmp, Psma3, Rnf114, Sec13, , Uba6, Ube2v2, and Vamp3 | Cyba Psmd13, Psmd14, Rbx1, Snap23 and Ube3a | 13 |
| **Cytokine and chemokine signaling** | Cotl1, H2-Aa, Hmgb1, Hnrnpa2b1, Hnrnpf, Psma3 and Rps6ka5 | Psmd13, Psmd14, Rbx1, Sell, Stxbp2 and Tnfrsf13c | 13 |
| **Signaling by the B cell receptor BCR** | Cd19, CLAM, Itpr2, Mzb1, Nfatc1, and Psma3, | Psmd13 and Psmd14 | 8 |
| **Autophagy** | Aimp2, Bak1, Elmo1, Hmgb1, and Lamtor1 | Cdk1, Mtmr3 and Rbx1 | 8 |
| **Fc epsilon receptor (FCERI) signaling** | CLAM, Itpr2, Psma3 and Rasgrp2 | Fcer2, Lyn, Psmd13 and Psmd14 | 8 |
| **B cell proliferation** | Cd81, Ighdm, and Rela | Tnfrsf13c and Ly86 | 5 |
| **Fcgamma receptor (FCGR) dependent phagocytosis** | Elmo1, Itpr2 and Was |  | 3 |
| **MHC class II antigen presentation** | H2-Aa and Sec13 | Dync1li1 | 3 |
