# Supplementary material for: Deficiency of STING Promotes Collagen-Specific Antibody Production and B Cell Survival in Collagen-Induced Arthritis
Source: Front Immunol. 2020 Jun 3;11:1101. doi: 10.3389/fimmu.2020.01101 (PMC7283782; doi:10.3389/fimmu.2020.01101)
Supplement: Supplementary file 3 [file Data_Sheet_1.docx]

Supplementary Material

# Supplementary Data

The Supplementary Material for this article can be found online.

# Supplementary Figures and Tables

## Supplementary Figures

Supplementary Figure 1. Activation of STING mediated B cell death.


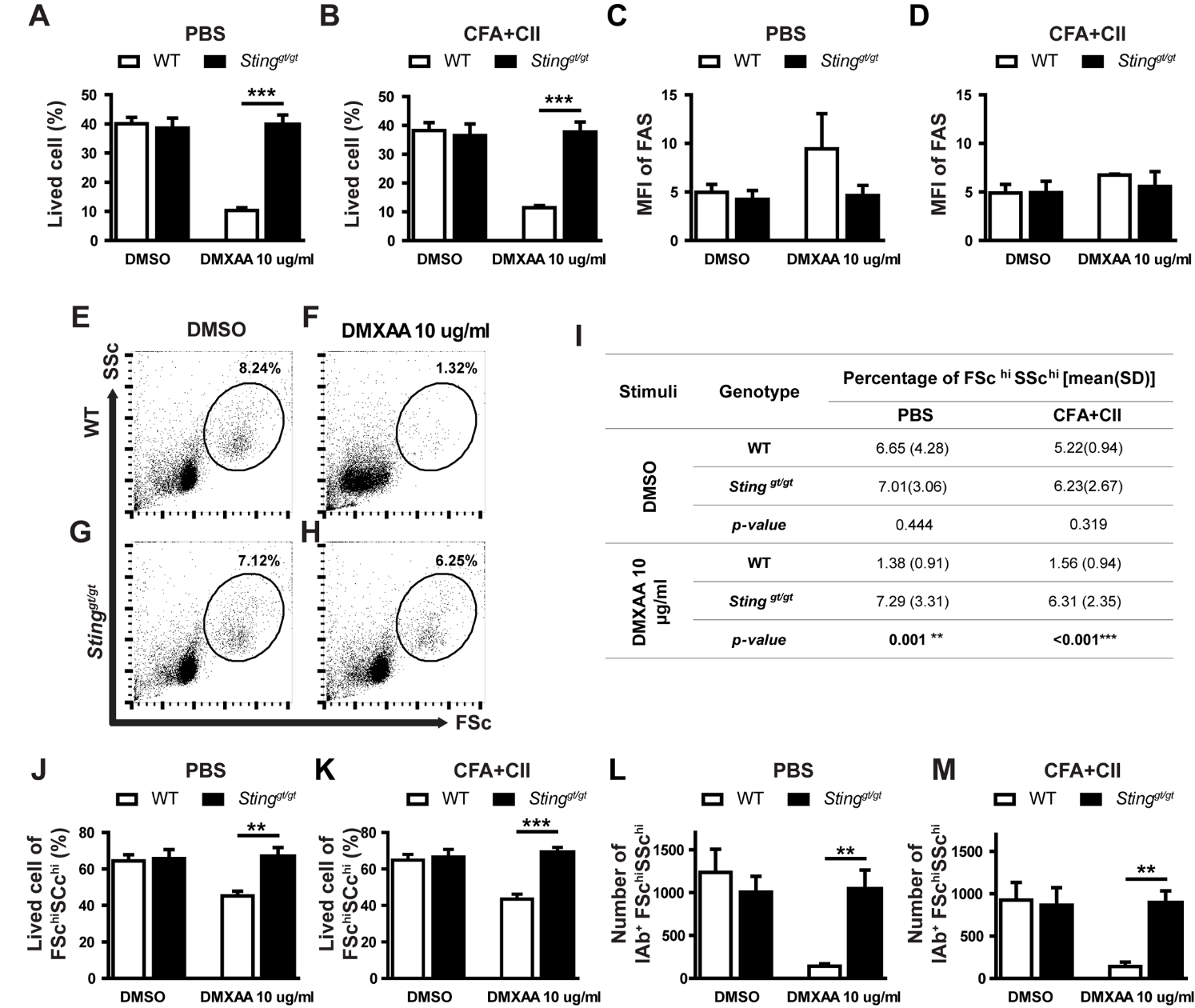


**Supplementary Figure 1.** **Activation of STING mediated B cell death.**

The isolated B cells from the spleens of immunized mice (WT and *Sting^gt/gt^* ) were cultured with DMSO control or DMXAA for 6 hours and analyzed by flow cytometry (A-M). (A-B) Percentage of B cell viability of (A) PBS and (B) CFA+CII injection group. (C-D) Mean fluorescence intensity (MFI) of FAS on B cells from (A) PBS and (B) CFA+CII injection group. (E-H) Representative plots from the PBS injection group show FSC and SSc. The gated areas show FSc^hi^SSc^hi^ B cells population. The isolated B cells from WT and *Sting^gt/gt^* mice were cultured with (E, G) DMSO and (F, H) DMXAA. (I) Data showed the percentage of FSc^hi^SSc^hi^ B cells isolated from the immunized WT and *Sting^gt/gt^* mice after incubation with DMSO and DMXAA. (J-M) Flow cytometry analysis showed (J-K) the percentage of viable cells in FSc^hi^SSc^hi^ B cells and (L-M) the number of IAb^+^FSc^hi^SSc^hi^ B cells from the mice injected with PBS and CFA+CII. Data are shown as mean + SEM (N=3-6 mice per group; *p < 0.05, **p<0.01, and ***p<0.001).

## Supplementary Table

Supplement Table 1: Biological process of RNA expression from Microarrays data

Supplement Table 2: Biological process of B cell protein from LC-MS/MS
